# A Study of Tooth Development, with Special Reference to the Formation of Enamel

**Published:** 1885-06

**Authors:** 


### A STUDY OF TOOTH DEVELOPMENT WITH SPECIAL REFERENCE TO THE FORMATION OF ENAMEL.

---

The following is a condensed report of a paper read by Henry Beales, Jr., M. D., before the Odontological Society of Pennsylvania, April 4, 1885. Professor S. H. Guilford presiding.

The paper was introduced by a description of the first morphological manifestations of tooth development, as constituted in the formation of what is known as the "dentinal ridge" and "lamina," drawings being exhibited to assist in a more thorough comprehension of the subject. The first illustration was taken from an embryo *before* dental formation is perceptible, and represented the mucus lining of the mouth derived from the epiblast and epithelial in character, the mesoblastic structure in type of connective tissue, and, separating the two, a thin layer, or basement membrane. Particular attention was called to the fact that the upper portion of the section, above the basement membrane, under a low power simulates in appearance columnar epithelium, and that this illusion is dissipated by a view of the so-called columnar layer with a one-fifth objective, under which it is seen that it is an area composed of epithelial cells, which by their relative arrangement are sub-divided into a superior and inferior zone, the cells occupying the upper or superior zone being more super-imposed than those below; also that the nuclei of these super-imposed cells are less distinct than those in the lower area. From this premise it is inferred that the epiblastic layer for the superior zone is concerned in the evolution of one element, and the inferior in another. Proceeding, the next diagram represented the dentinal ridge and lamina, the cells composing the superior zone having undergone marked proliferation, and those of the inferior zone having sunken into the mesoblastic area, the V-shaped invasion being a purely physiological action. The next noticeable step is the branching out, from an arm of the V-shaped lamina, of the so-called Cord. As this is achieved the zone manifests a trivial, but at the same time a definite, demonstrable, and undeniable change. The cells of the superior epiblastic zone are now *transverse*, as far as the isthmus of the cord; the cells contained in its expanding extremity not having undergone the transverse change, but being in a state of local functional activity, the result is the formation of a stirrup-like growth, the special vital endowment being possessed more particularly by those cells which occupy the position corresponding with the base.

The next diagram submitted represented a section showing the first morphological developments, bearing directly upon the facts determining the hypothesis submitted, and attention was called to the obliteration of the neck of the cord from the arm of the V-shaped lamina; the stirrup-shaped development of the extremity of the cord; the transverse cells, the incipient change prior to the formation of a reticulum; the mesoblastic area and seat of development of the dentinal bulb, the more active area corresponding with the apex of the future tooth; the sac of follicle, and the basement membrane. The different appearance presented at the base of the stirrup from the sides or arms, can only be differentiated by a comparatively high power objective—one-fifth or one-eighth. The special feature to which reference is made is that at the upper area of the base the cells are proliferating, and present marked activity at a point corresponding with the situation of the future apex; that the central area is relatively inactive, and that the inferior area, like the superior, is active. The base of the stirrup thus assumes a tri-zonal area of special physiological activity. Under a one-half inch objective this proliferating base presents itself as a simple thickening, which from its trivial appearance has been universally ignored, or probably regarded as an accidental condition dependent upon manipulation. A point to be noted is that when the adult tooth shall have been developed, the base of the stirrup, described as the tri-zonal area, will constitute its covering *from apex to neck.* An older section enabled a more clear tracing of the facts presented to be effected. The changes manifested were the mesoblastic formation of the apex of the bulb, beginning to indent the base of the stirrup; the central cells derived from the superior zone of the original mucous membrane or epiblastic covering, and their development into the reticulum, which is claimed to be the enamel organ, or at least its secreting element. The inferior zone is especially developed, and will be the structure immediately super-imposed upon the dentinal bulb in the adult tooth; its cellular quantity is more appreciable at the apex, and diminishes progressively to the point which will be the neck of the tooth. The middle zone is of uniform thickness, no deeper at the apex than at the neck. The superior zone is not particularly developed, and is simply distributed over the apex, being a little deeper at the latter point. The processes continuing are the development of the dentinal bulb, which pushes up the base of the stirrup, separating the reticulum into the lateral lobes, crescentic in contour, and applied to each side of the tooth from apex to base, and the appearance of blood vessels which permeate the dentinal bulb in great number. These vessels reach the extreme limits of the bulb, and serve as tributaries to convey pabulum to this component element of the future tooth. The tri-zonal base has also undergone marked change, and notably the inferior zone. The superior zone is simply, as before, spread over the surface of the middle zone. The latter is now a genuine columnar layer of epithelium, of uniform thickness from apex to cervix, while the inferior zone is thickest at the apex, and thinnest at the cervix. At the peripheral border of the dentinal bulb, where in earlier sections rounded cells existed, there are now developed elongated cells, which possess polar prolongations entering into the area which will be occupied by future dentine. Prior to the development of blood vessels in the bulb there was no trace of enamel, dentine, or odontoblasts, but simply an arrangement of the cells into their respective positions, where they could most effectively elaborate the morphological functions with which they were endowed. The next diagram exhibited, amongst other points, the fact that *at the apex where the enamel is developed the greatest, the reticulum is least.* The significance of this is of twofold force, when the fact is recalled that this point will very soon penetrate the reticulum, and that the enamel at this time is *not* formed, but only beginning, and is completed at a future time, *i. e.*, when the reticulum will have been situated near the base or root portion of the follicle. If the reticulum secretes enamel salts, how can enamel develop at a portion of the tooth remote from that reticulum, and why is the root of the tooth not covered with enamel also? Or, in other words, if this middle zone constitutes the ameloblasts, and elaborates the enamel from the material supplied by a secretive function of the reticulum, why is not enamel formed or deposited at that portion of the dentine which will later develop and constitute the root, especially as the ameloblastic layer and reticulum occupy a position in immediate juxtaposition to the root.

A study of an older slide disclosed the fact that the inferior portion of the tri-zonal base, now decidedly developed, *ceases to exist at the neck of the tooth,* which endorses the significance of the previous remark, that the inferior zone extended from apex to cervix dentis *only*, and explains the necessity for a careful study and tracing of the tri-zonal area.

The next diagram represented a section taken from an eight months' human fœtus, and showed the reticulum occupying a basal position close to the root, not having kept pace with the development of the tooth. As the root develops and pushes the body of the tooth upward, the reticulum will occupy the position of the root and *form the cementum.* The inferior zone is now seen as well developed enamel, its apical development and steadily diminishing thickness until the point of cessation at the cervix dentis, being conspicuous. The so-called ameloblastic layer appears as a single uniform line of columnar cells, extending well *below* the point at which enamel ceases to exist.

From this study the theory regarding tooth development, and especially the formation of enamel, may be formulated as follows, viz.: Teeth evolve from the epiblast and mesoblast; the enamel and cementum are derivatives of the epiblast, the dentine of the mesoblast. Enamel is evolved from the inferior zone of the tri-zonal base described. The inferior zone receives its material for elaboration from the pulp, by means of the polar prolongations of the odontoblasts, which traverse the dentinal tubules and are in direct apposition to, and in many instances continuous with, the enamel rods.

After the reading of the paper, discussion of the subject was invited, but before doing so it was suggested that an adjournment take place to examine the sections under the microscope. On reassembling, remarks *pro* or *con* were again asked for, Dr. Beates having during the interval exhibited his specimens and further explained the theories advanced. But either from modesty, or inability to cope with a subject at present engaging the attention of the leading men of the profession, and occupying much space in all dental journals, the subject failed to elicit argumentative remark from anyone present, although points had been evolved directly antagonistic to the theories held and taught in our dental schools.

In proposing a vote of thanks, Dr. Tees remarked upon the great indebtedness of the dental profession to Dr. Beates, for presenting the subject in the manner he had, especially as the study was one which did not come under his immediate professional attention.

Professor Guilford endorsed this remark, and stated that the Society was much interested in the paper, but their meetings were usually occupied with practical subjects, and the points advanced, being purely theoretical, accounted in a measure for the absence of discussion by the members present. The subject was a comparatively new one, consequently they were not familiar with it.

Professor Truman wished to correct this statement. He believed this subject had been brought forward many years since by the elder Tomes, and had been before the profession ever since, agitated by Magitot, Dean, Flower, Williams and others.

